# Molecular evolution of the *VP1, VP2*, and *VP3* genes in human rhinovirus species C

**DOI:** 10.1038/srep08185

**Published:** 2015-02-02

**Authors:** Makoto Kuroda, Shoichi Niwa, Tsuyoshi Sekizuka, Hiroyuki Tsukagoshi, Masaru Yokoyama, Akihide Ryo, Hironori Sato, Naoko Kiyota, Masahiro Noda, Kunihisa Kozawa, Komei Shirabe, Takashi Kusaka, Naoki Shimojo, Shunji Hasegawa, Kazuko Sugai, Masatsugu Obuchi, Masato Tashiro, Kazunori Oishi, Haruyuki Ishii, Hirokazu Kimura

**Affiliations:** 1 Pathogen Genomics Center, National Institute of Infectious Diseases, 1-23-1 Toyama, Shinjuku-ku, Tokyo 162-8640, Japan; 2 Gunma Prefectural Institute of Public Health and Environmental Sciences, 378 Kamioki-machi, Maebashi-shi, Gunma 371-0052, Japan; 3 Department of Molecular Biodefence Research, Yokohama City University Graduate School of Medicine, 3-9 Fukuura, Kanazawa-ku, Yokohama-shi, Kanagawa 236-0004, Japan; 4 Kumamoto Prefectural Institute of Public Health and Environmental Sciences, 1240-1, Kurisaki-machi, Uto-shi, Kumamoto 869-0425, Japan; 5 Infectious Disease Surveillance Center, National Institute of Infectious Diseases, 4-7-1 Gakuen, Musashimurayama-shi, Tokyo 208-0011, Japan; 6 Yamaguchi Prefectural Institute of Public Health and Environment, 2-57-6 Aoi, Yamaguchi-shi, Yamaguchi 753-082, Japan; 7 Maternal Perinatal Center, Faculty of Medicine, Kagawa University, 1750-1 Ikenobe, Miki-cho, Kita-gun, Kagawa 761-0793, Japan; 8 Department of Pediatrics, Graduate School of Medicine, Chiba University, 1-8-1 Inohana, Chuo-ku, Chiba-shi, Chiba 260-8670, Japan; 9 Department of Pediatrics, Yamaguchi University Graduate School of Medicine, 1-1-1 Minami-kogushi, Ube-shi, Yamaguchi 755-8505, Japan; 10 Department of Pediatrics, National Hospital Organization Yokohama Medical Center, 3-60-2 Harajuku, Totsuka-ku, Yokohama, Kanagawa 245-8575, Japan; 11 Toyama Institute of Health, 17-1 Nakataikoyama, Imizu-shi, Toyama 939-0363, Japan; 12 Influenza virus Research Center, National Institute of Infectious Diseases, 4-7-1 Gakuen, Musashimurayama-shi, Tokyo 208-0011, Japan; 13 Department of Respiratory Medicine, Kyorin University, School of Medicine, 6-20-2 Shinkawa, Mitaka-shi, Tokyo 181-8611, Japan

## Abstract

Human rhinovirus species C (HRV-C) was recently discovered, and this virus has been associated with various acute respiratory illnesses (ARI). However, the molecular evolution of the major antigens of this virus, including VP1, VP2, and VP3, is unknown. Thus, we performed complete *VP1, VP2,* and *VP3* gene analyses of 139 clinical HRV-C strains using RT-PCR with newly designed primer sets and next-generation sequencing. We assessed the time-scale evolution and evolutionary rate of these genes using the Bayesian Markov chain Monte Carlo method. In addition, we calculated the pairwise distance and confirmed the positive/negative selection sites in these genes. The phylogenetic trees showed that the HRV-C strains analyzed using these genes could be dated back approximately 400 to 900 years, and these strains exhibited high evolutionary rates (1.35 to 3.74 × 10^−3^ substitutions/site/year). Many genotypes (>40) were confirmed in the phylogenetic trees. Furthermore, no positively selected site was found in the VP1, VP2, and VP3 protein. Molecular modeling analysis combined with variation analysis suggested that the exterior surfaces of the VP1, VP2 and VP3 proteins are rich in loops and are highly variable. These results suggested that HRV-C may have an old history and unique antigenicity as an agent of various ARI.

Human rhinovirus (HRV) belongs to the genus *Enterovirus* and the family *Picornaviridae*. HRV is the common causative agent of acute respiratory illness (ARI), such as the common cold, bronchiolitis, and pneumonia^1^. HRV is classified into three species: HRV-A, -B, and -C^1^. HRV-C was recently discovered, and this virus is not easily cultured by conventional methods^2^. Additionally, molecular epidemiological studies have suggested that HRV-C may have wide genetic diversity^3^. However, their virology is not exactly understood.

In general, enteroviruses, including HRV, possess the structural viral proteins (VP) 1 to 4. These are role-sharing proteins, and of these proteins, VP1, VP2, and VP3 are both major structural proteins and essential antigens^1^, whereas VP4 is a scaffold protein of the viral capsid^4^. Additionally, the divergence of the VP1, VP2, and VP3 proteins is linked to the antigenicity of the various enteroviruses, resulting in different serotypes^5^. However, the essential knowledge of antigenicity regarding HRV-C is poorly understood.

Viral molecular evolution is different for each virus, and it may be responsible for their genome size and genome polarity^6^. The positive pressure of the host may lead to changes in the selection site, leading to changes in antigenicity faster than if spontaneous mutations had occurred^7^. Therefore, such factors should be considered when we refer to the evolution of viral genes^7^.

Recent phylogenetic technologies, including the Bayesian Markov chain Monte Carlo (MCMC) method, enable us to perform cluster analysis and time scale evolution of the various viral genes^8^. For example, Tsukagoshi *et al*. showed that the evolution rate of the C-terminal 3rd hypervariable region in the glycoprotein gene, which is a major antigen of respiratory syncytial virus (RSV), was rapid, as was the hemagglutinin (*H*) gene in the influenza virus subtype AH1^9^. Furthermore, Mizuta *et al*. showed that the evolutionary rate of the hemagglutinin-neuraminidase (*HN*) gene in human parainfluenza virus type 1 was nearly equal to the *H* gene of the measles virus^10^,^11^. However, the molecular evolution of the major structural proteins, such as the VP1, VP2, and VP3 proteins, in HRV-C is still unknown due to the wide genetic divergence of the VP protein coding regions in HRV-C; this divergence makes it hard to analyze their genetic properties. We were able to overcome that difficulty by analyzing the major antigen coding regions of HRV-C using next-generation sequencing (NGS) with thoroughly prepared primers. Additionally, we calculated the structure of the VP1, VP2 and VP3 proteins *in silico*. Using these advanced methods, we studied the molecular evolution of the *VP1, VP2,* and *VP3* genes in HRV-C (139 strains) detected in various ARI patients.

## Results

### HRV-C specific RT-PCR and amplicon sequencing using NGS

To amplify the highly variable HRV-C genomic RNA by RT-PCR, suitable degenerate primers were designed using the following procedures. Publicly available HRV-C complete genome sequences (23 strains) were aligned by MUSCLE in the MEGA 5.0 software, and a potential primer site was determined for a possible common reverse primer to lower variation. The availability of the degenerate primer HRV-C_6410R was evaluated with 8 randomly selected clinical specimens; a 5.8-kb RT-PCR product was observed for all 8 tested samples, suggesting that the primer could efficiently amplify the long range of the HRV-C genome (Suppl. Fig. S1). The amplicons theoretically contain an incomplete *VP4* coding region, complete *VP2* to *3C* regions, and an incomplete *3D* region. To investigate the molecular evolution of the major antigens, we focused on the analyses of the *VP1*, *VP2* and *VP3* genes.

Using the above-described a primer set for PCR amplification, 187 amplicons were obtained and the amplicons were *de novo* sequenced using NGS with multiple indexing (~100,000 reads for each sample ID); these sequences were then assembled into the respective 5.8 kb HRV-C contig sequences. Homology searches of these contigs suggested that 139 of the 187 amplicons (74.3%) derived from clinical specimens were able to analyze as HRV-C strains. To characterize the genetic evolution and immunogenicity of HRV-C, the potential coding regions of the *VP1*, *VP2* and *VP3* genes in the obtained contigs were aligned with the reference HRV-C sequences.

### Phylogenetic analysis of the VP1 gene in HRV-C

**Figure 1:**
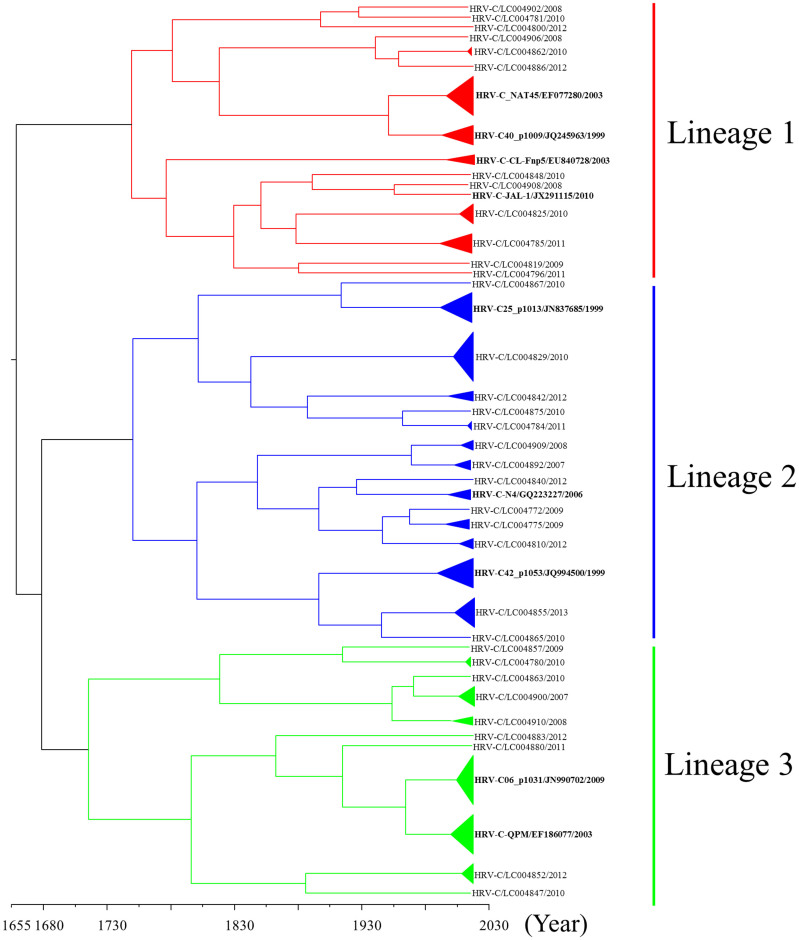
Phylogenetic tree of the *VP1* gene in HRV-C constructed using the Bayesian MCMC method. A phylogenetic tree based on *VP1* gene sequences. In the tree, the triangle size expresses the number of strains, while only one representative strain is listed for each genotype. The strains are presented as follows: virus species/GenBank accession no./year. The GenBank accession numbers of the reference strains are indicated in bold letters. The scale bar represents the unit of time (year).

We analyzed the full sequence of the *VP1* coding region (804–846 nt) in 139 strains of HRV-C detected in ARI patients. Using the Bayesian MCMC method, we constructed a phylogenetic tree with time scale evolution (Fig. 1). First, the present strains comprised three major lineages. When we genotyped the samples using 13% nucleotide divergence^3^,^12^, 44 genotypes were confirmed. Lineages 1, 2, and 3 contained 16, 16, and 12 genotypes, respectively. The most recent common ancestor (tMRCA) of all of the strains was found in 1652 (95% highest posterior density (HPD) 1522 to 1769). The lineages 1, 2, and 3 were dated back to 1744 (95% HPD 1640 to 1832), 1745 (95% HPD 1642 to 1832), and 1710 (95% HPD 1594 to 1812), respectively. The evolution rate was 3.48 × 10^−3^ substitutions/site/year (95% HPD 2.36 × 10^−3^ to 4.60 × 10^−3^). The results suggested that the *VP1* gene in HRV-C showed a wide range of genetic divergence with a high rate of evolution.

### Phylogenetic analysis of the VP2 gene in HRV-C

**Figure 2:**
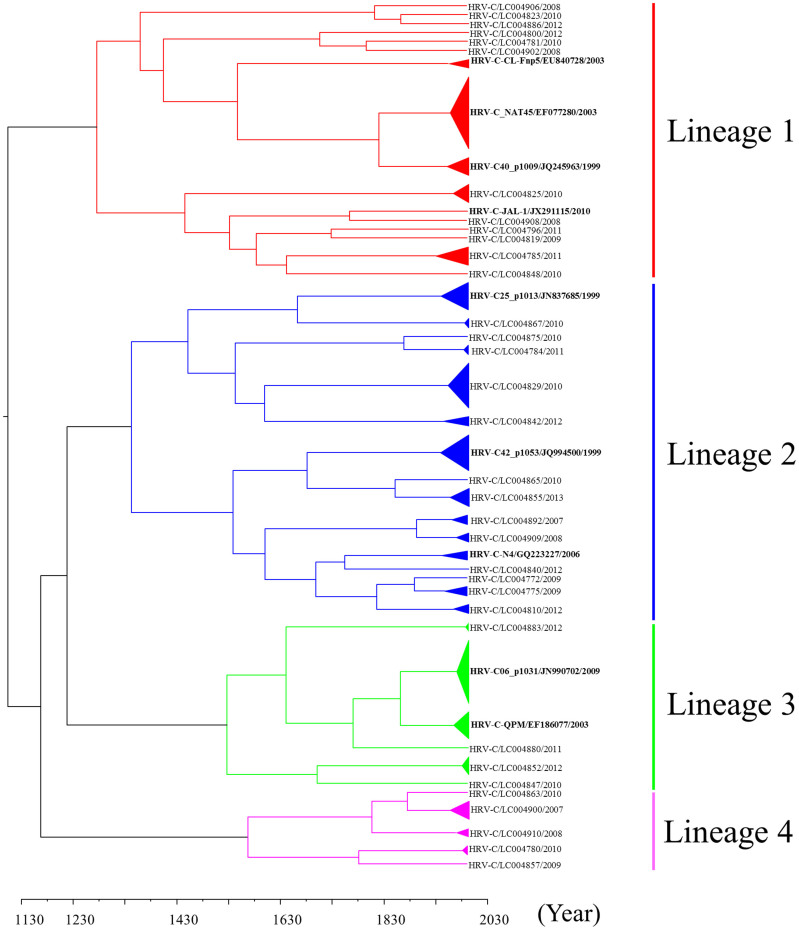
Phylogenetic tree of the *VP2* gene in HRV-C constructed using the Bayesian MCMC method. A phylogenetic tree based on *VP2* gene sequences. In the tree, the triangle size expresses the number of strains, while only one representative strain is listed for each genotype. The strains are presented as follows: virus species/GenBank accession no./year. The GenBank accession numbers of the reference strains are indicated in bold letters. The scale bar represents the unit of time (year).

As well as *VP1* gene, we analyzed the full sequence of the *VP2* coding region (783–816 nt) in 139 strains and a phylogenetic tree was constructed with time scale evolution (Fig. 1B). First, the present strains comprised four major lineages, similar to the phylogenetic tree based on the full *VP1* coding region (Fig. 2). When we genotyped the strains using 13% nucleotide divergence^3^,^12^, 43 genotypes were confirmed. Lineages 1, 2, 3, and 4 contained 16, 16, 6, and 5 genotypes, respectively. tMRCA of all of the strains was found in 1125 (95% HPD 570 to 1548). Lineages 1, 2, 3, and 4 were dated back to 1294 (95% HPD 853 to 1643), 1361 (95% HPD 955 to 1681), 1545 (95% HPD 1244 to 1775), and 1586 (95% HPD 1312 to 1803), respectively. The evolution rate was 1.35 × 10^−3^ substitutions/site/year (95% HPD 2.12 × 10^−3^ to 6.46 × 10^−4^). The results suggested that HRV-C *VP2* gene of the present strains had a wide range of genetic divergence with a high rate of evolution as well as their *VP1* gene.

### Phylogenetic analysis of the VP3 gene in HRV-C

**Figure 3:**
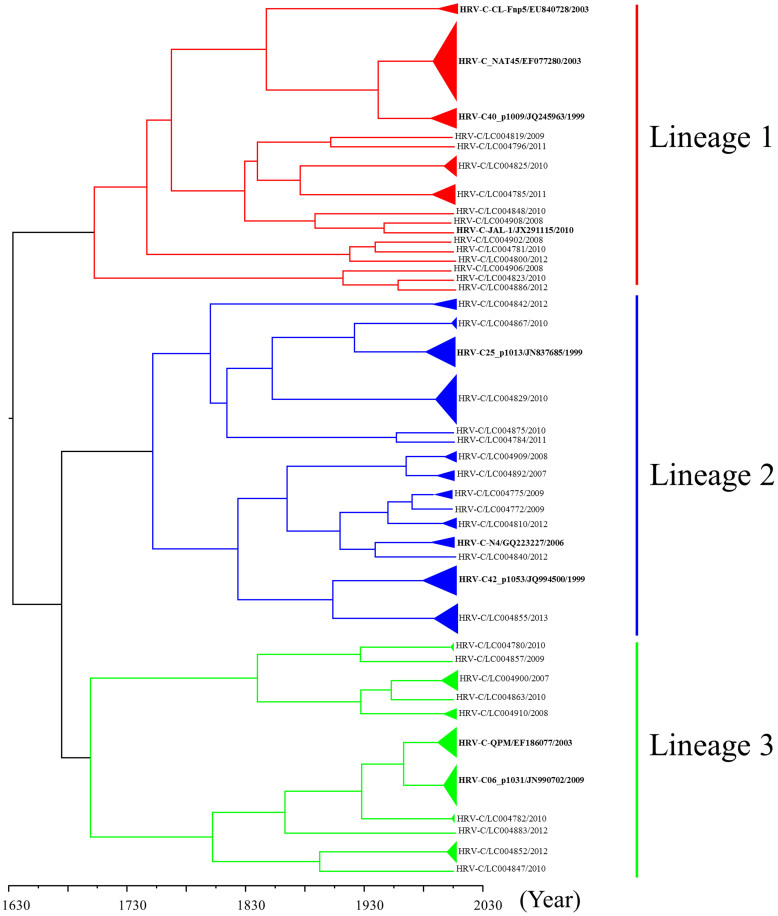
Phylogenetic tree of the *VP3* gene in HRV-C constructed using the Bayesian MCMC method. A phylogenetic tree based on *VP3* gene sequences. In the tree, the triangle size expresses the number of strains, while only one representative strain is listed for each genotype. The strains are presented as follows: virus species/GenBank accession no./year. The GenBank accession numbers of the reference strains are indicated in bold letters. The scale bar represents the unit of time (year).

We analyzed the full sequence of the *VP3* coding region (699–717 nt) in 139 strains of HRV-C. We constructed a phylogenetic tree with time scale evolution as well as *VP1* and *VP2* genes (Fig. 3). First, the present strains comprised three major lineages, similar to the phylogenetic tree based on the full *VP1* coding region (Fig. 3). When we genotyped the samples using 13% nucleotide divergence^3^,^12^, 42 genotypes were confirmed. Lineages 1, 2, and 3 contained 16, 15, and 11 genotypes, respectively. tMRCA of all of the strains was found in 1628 (95% HPD 1494 to 1746). Lineages 1, 2, and 3 were dated back to 1698 (95% HPD 1579 to 1797), 1749 (95% HPD 1654 to 1829), and 1695 (95% HPD 1581 to 1796), respectively. The evolution rate was 3.74 × 10^−3^ substitutions/site/year (95% HPD 2.63 × 10^−3^ to 4.91 × 10^−3^). The results suggested that the *VP3* gene in HRV-C showed a wide range of genetic divergence with a high rate of evolution as well as the *VP1 and VP2* genes.

### Genetic divergence of the VP1 gene in HRV-C

**Figure 4:**
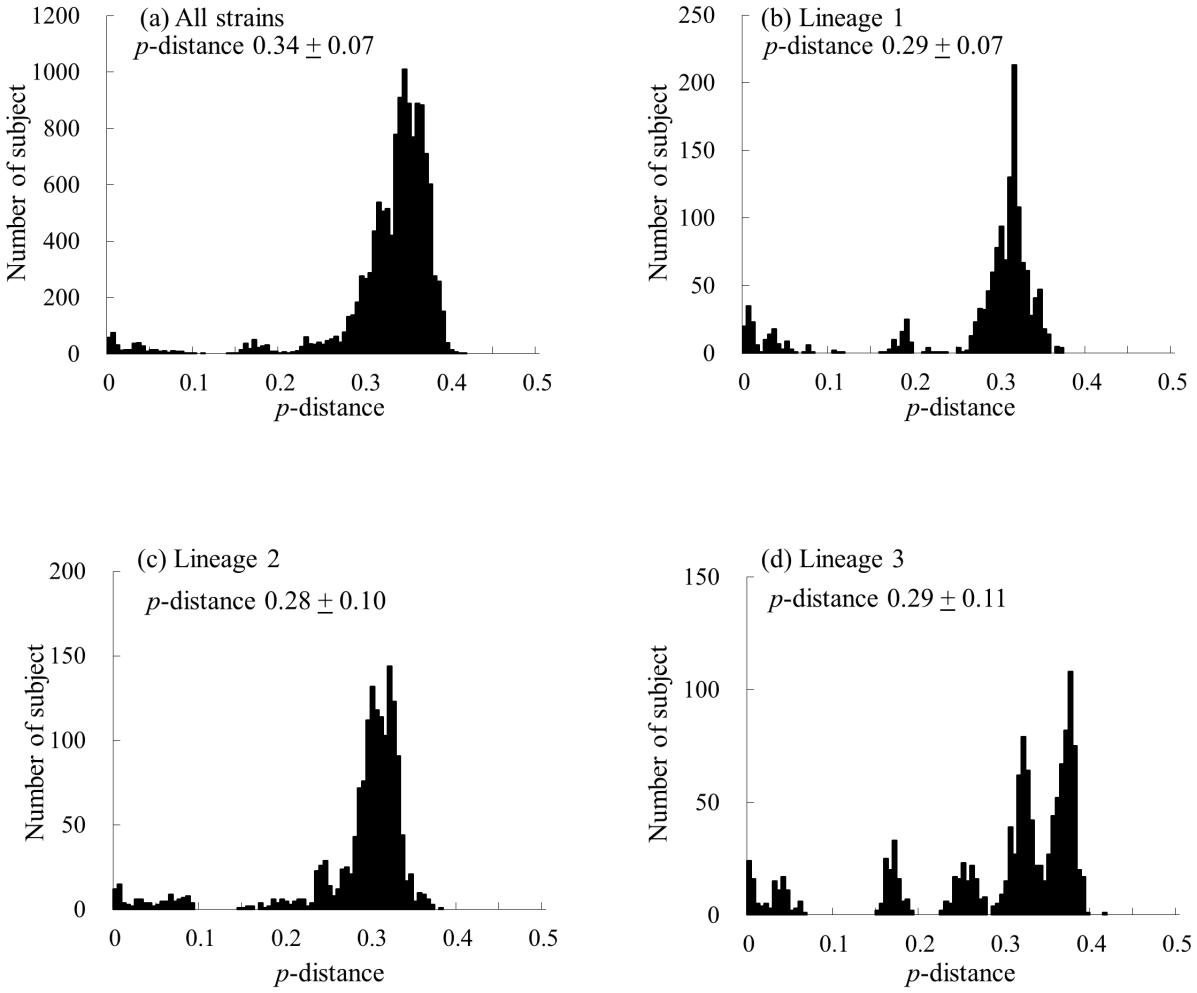
The distribution of the pairwise interspecies distances based on the nucleotide sequences of the *VP1* gene. (a) The distribution of all strains. (b–d) The distributions of the pairwise distances for each lineage (lineages 1–3).

We calculated the nucleotide identity of the *VP1* gene in 139 strains of HRV-C. Among the present strains, the nucleotide identity was 60.2 to 100%, and the deduced amino acid identity was 58.5 to 100%. Among the present strains in lineages 1, 2, and 3, the nucleotide identity was 65.4–100%, 63.0–100%, and 61.2–100%, respectively, while among the present strains, the deduced amino acid identity was 68.5–100%, 67.0–100%, and 59.0–100%, respectively. Furthermore, we calculated the *p*-distance of the present strains (Fig. 4 (a)). As a result, the *p*-distance of all strains was 0.34 ± 0.07 (mean ± standard deviation, SD) at the nucleotide level and 0.32 ± 0.08 at the deduced amino acid level. Among the present strains in lineages 1, 2, and 3, the values were 0.29 ± 0.07, 0.28 ± 0.10, and 0.29 ± 0.11 at the nucleotide level, respectively (Fig. 4 (b–d)), and the deduced amino acid *p*-distance values were 0.25 ± 0.08, 0.23 ± 0.09, and 0.27 ± 0.12, respectively.

### Genetic divergence of the VP2 gene in HRV-C

**Figure 5:**
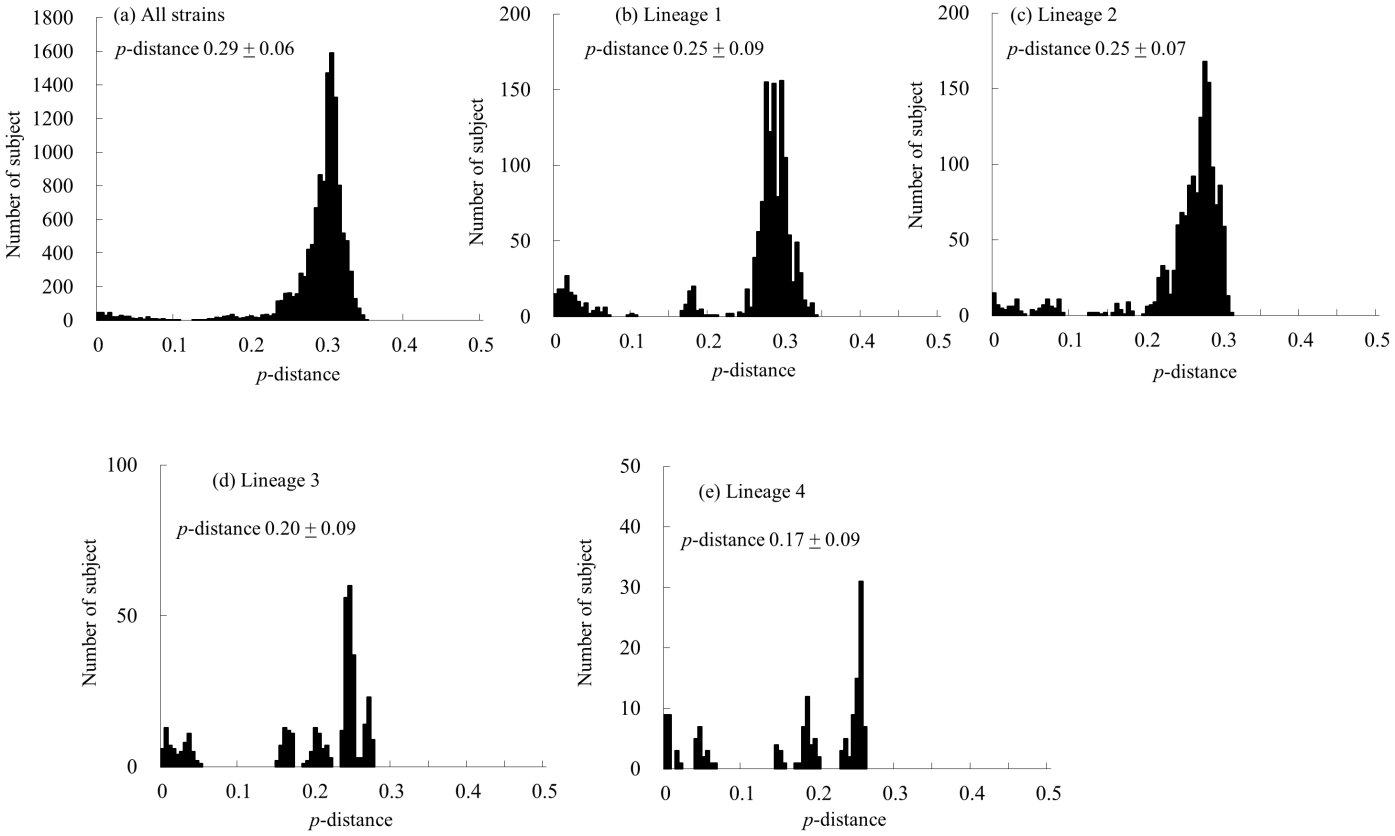
The distribution of the pairwise interspecies distances based on the nucleotide sequences of the *VP2* gene. (a) The distribution of all strains. (b–e) The distributions of the pairwise distances for each lineage (lineages 1–4).

We calculated the nucleotide identity of the *VP2* gene in 139 strains of HRV-C. The nucleotide identity was 66.2 to 100%, and the deduced amino acid identity was 70.7 to 100%. Lineages 1, 2, 3 and 4, the nucleotide identity was 67.0–100%, 69.3–100%, 72.2–100% and 73.8–100%, respectively, while the deduced amino acid identity was 71.3–100%, 75.7–100%, 82.3–100%, and 82.6–100%, respectively. We also calculated the *p*-distance of the present strains (Fig. 5 (a)). The *p*-distance of all strains was 0.29 ± 0.06 (mean ± standard deviation, SD) at the nucleotide level and 0.23 ± 0.06 at the deduced amino acid level. Lineages 1, 2, 3, and 4, the values were 0.25 ± 0.09, 0.25 ± 0.07, 0.20 ± 0.09 and 0.17 ± 0.09 at the nucleotide level, respectively (Fig. 5 (b–e)), and the deduced amino acid *p*-distance values were 0.20 ± 0.08, 0.18 ± 0.06, 0.10 ± 0.06 and 0.09 ± 0.07, respectively.

### Genetic divergence of the VP3 gene in HRV-C

**Figure 6:**
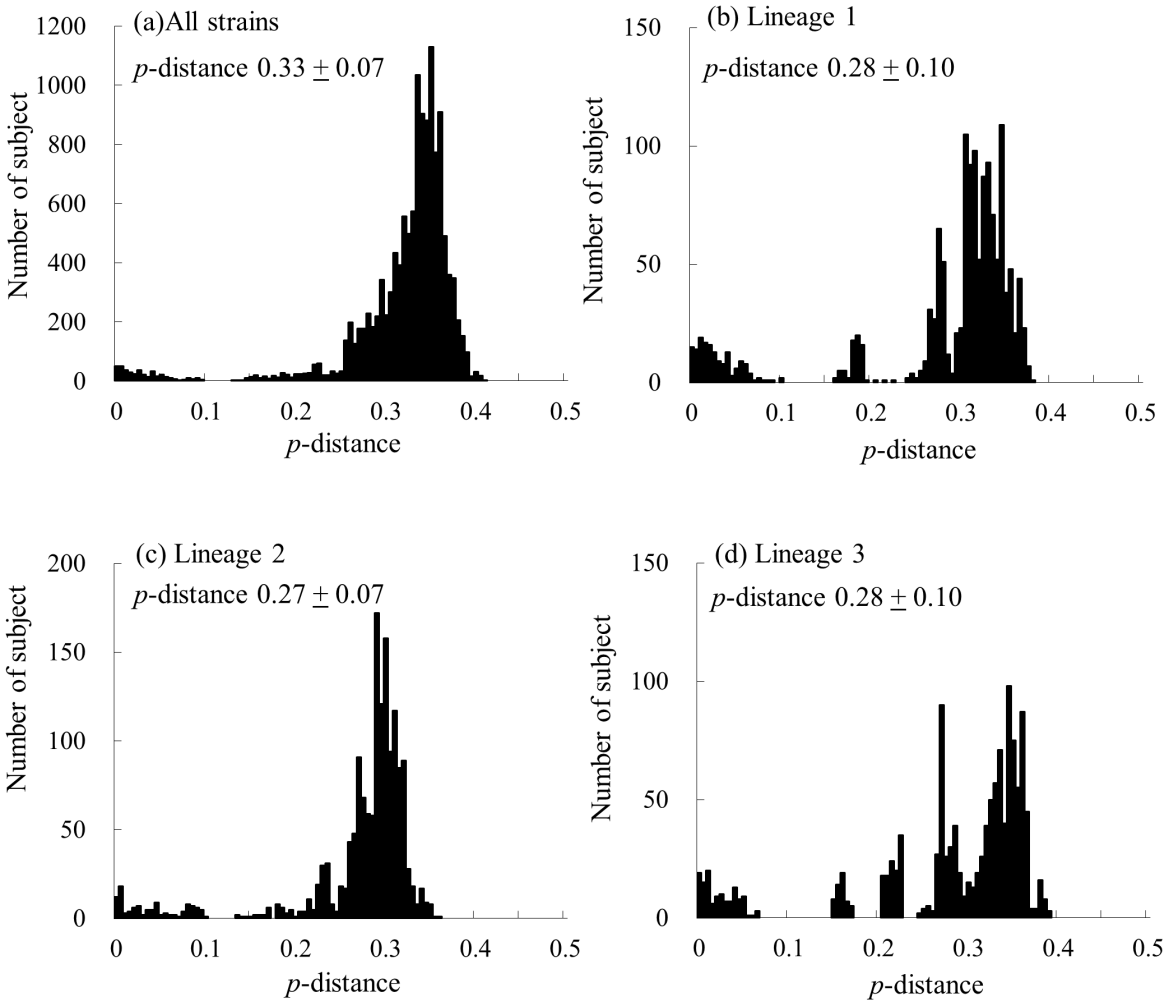
The distribution of the pairwise interspecies distances based on the nucleotide sequences of the *VP3* gene. (a) The distribution of all strains. (b–d) The distributions of the pairwise distances for each lineage (lineages 1–3).

We also calculated the nucleotide identity of the *VP3* gene in 139 strains of HRV-C. Among the present strains, the nucleotide identity was 59.4 to 100%, and the deduced amino acid identity was 58.3 to 100%. Lineages 1, 2, and 3, the nucleotide identity was 62.2–100%, 66.3–100%, and 61.9–100%, respectively, while the deduced amino acid identity was 65.4–100%, 70.7–100%, and 63.2–100%, respectively. Next, we calculated the *p*-distance of the present strains (Fig. 6 (a)). The *p*-distance of all strains was 0.33 ± 0.07 (mean ± SD) at the nucleotide level and 0.30 ± 0.08 at the deduced amino acid level. Among the present strains in lineages 1, 2, and 3, the values were 0.28 ± 0.10, 0.27 ± 0.07, and 0.28 ± 0.10 at the nucleotide level, respectively (Fig. 6 (b–d)) and 0.23 ± 0.10, 0.21 ± 0.08, and 0.24 ± 0.11, respectively, at the deduced amino acid level.

### Simplot data and recombination analysis of the VP1, VP2 and VP3 genes in the present strains

**Figure 7:**
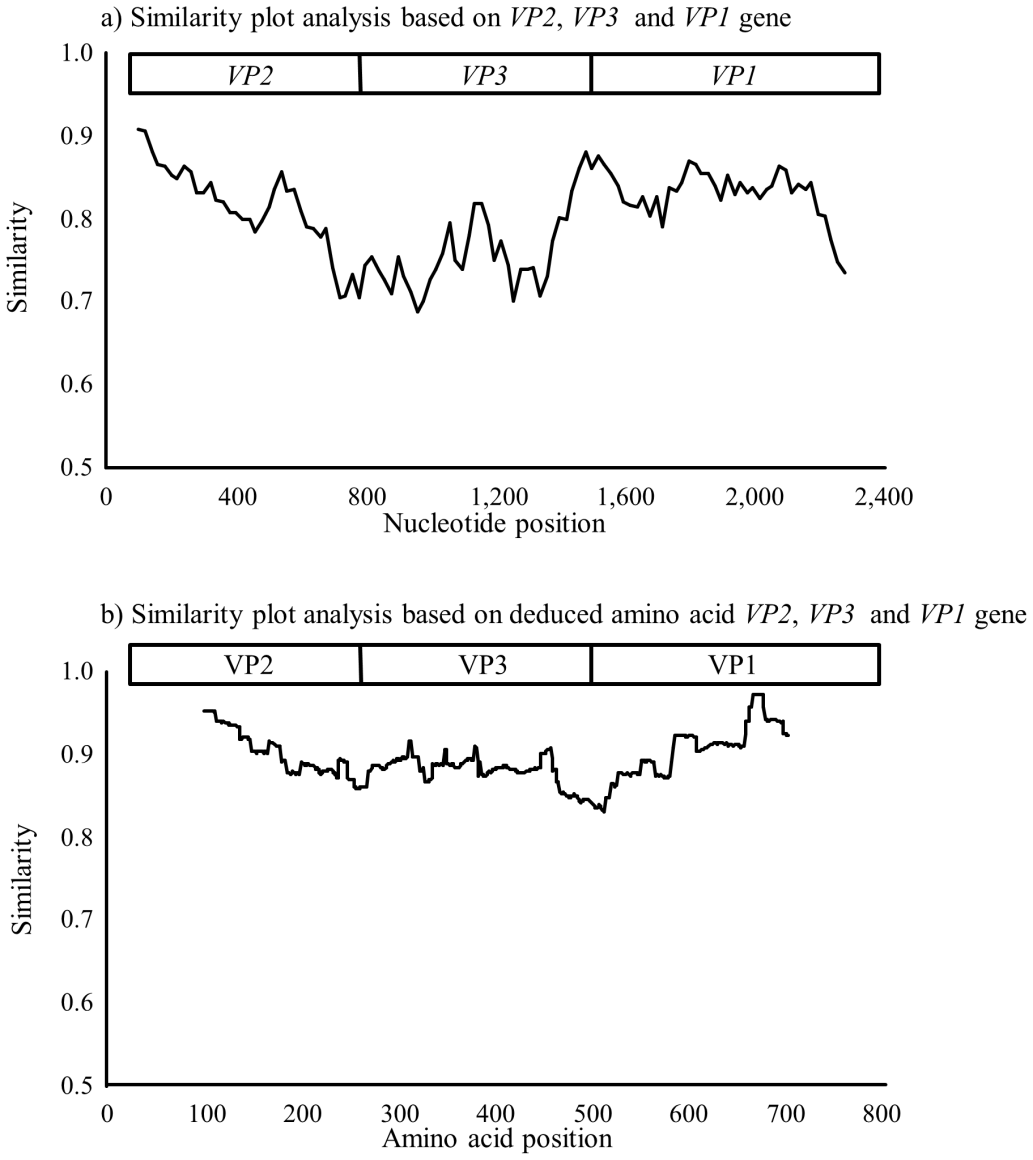
Characterization of the *VP1*, *VP2* and *VP3* genes in HRV-C. (a) Nucleotide similarity to HRV-QPM (prototype strain) was determined using SimPlot analysis. (b) Amino acid similarity to HRV-QPM (prototype strain) was determined using SimPlot analysis. The plots indicate the percentage of similarity to a 50% consensus sequence from each species' polyprotein compared to HRV-QPM.

We calculated the similarity of the nucleotide sequences of the *VP1, VP2* and *VP3* genes in the present strains and in a prototype strain (HRV-QPM strain). As shown in Fig. 7 (a), an overall high similarity of the *VP1* coding region was found when compared to the *VP3* coding region. The minimum similarities of the *VP2, VP3* and *VP1* genes were approximately 70, 68 and 72%, respectively. Additionally, the similarities of the 5′-terminal *VP3* coding region and the 3′-terminal *VP1* coding region were low (approximately 70%), whereas the similarities of the 3′-terminal *VP3* coding region and the 5′-terminal *VP1* coding region were high (approximately 80%). Additionally, we calculated the similarity of the deduced amino acid sequences of the *VP1* and *VP3* genes in the present strains and the prototype strain (HRV-QPM strain) (Fig. 7 (b)). The minimum similarities of the *VP2, VP3,* and *VP1* proteins were approximately 86, 83 and 87%, respectively. The Recombination Detection Program (RDP) found no evidence of a recombination event. The results suggested that high genetic divergence was found in the *VP3* coding region compared to the *VP2*, and *VP1* coding region.

### Association between positive selection sites and the possible structures of the VP1, VP2 and VP3 proteins

**Figure 8:**
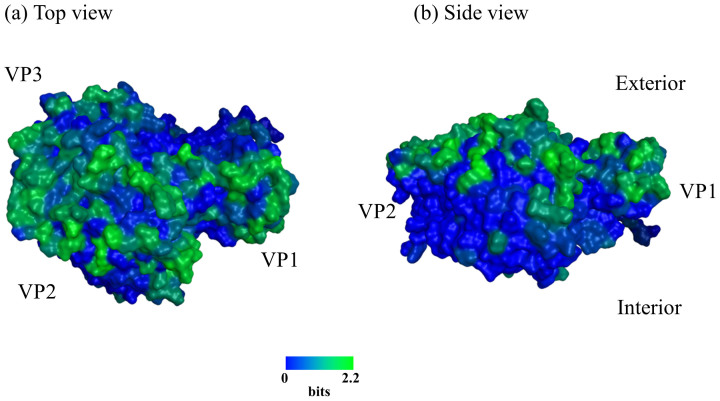
Shannon entropy scores expressed on the structural models of HRV-C VP1, VP2, and VP3 proteins. The HRV-C VP1, VP2, and VP3 models were constructed by homology modeling as described in Materials and Methods and were superimposed on the VP1, VP2, and VP3 in those of the HRV2 capsid (PDB code: 3VDD). The entropy scores are expressed on the HRV-C VP1, VP2, and VP3 models. (a) Top view. (b) Side view.

Using SLAC, FEL, and IFEL methods, we estimated the positive selection sites in the VP1, VP2, and VP3 proteins in HRV-C. No positively selected sites were detected in any position by any method, while many sites under negative selection (>100) were found. Next, we constructed a molecular model of the complex containing the HRV-C VP1, VP2, and VP3 proteins. The model showed that these proteins are rich in loop structures that are primarily positioned on the exterior surface of the capsid complex (Figs. 8 (a) and 8 (b)). Our Shannon entropy data show that these exterior loops of VP1, VP2 and VP3 are highly variable compared to the interior of the capsid within the HRV-C population analyzed in this study (Figs. 8 (a) and 8 (b)).

## Discussion

We studied the molecular evolution of the full length *VP1*, *VP2*, and *VP3* genes in HRV-C detected from ARI. Analysis of the full sequences of the major 3 viral protein genes in the current HRV-C strains was performed using NGS with an improved RT-PCR method. Time-scaled phylogenetic analysis with evolution rate for the genes was analyzed using the Bayesian MCMC method. Additionally, we constructed the VP1, VP2, and VP3 proteins using an *in silico* method. First, the phylogenetic trees based on the *VP1*, *VP2*, and *VP3* genes showed that the current HRV-C strains were classified into 3 or 4 major lineages, and these lineages were subdivided into many genotypes (>40). The most recent common ancestor (tMRCA) of all the strains based on the *VP1*, *VP2*, and *VP3* genes was found in the years 1652, 1125 and 1628, respectively. The evolution rates of both genes were fast. Similarity plot data showed high genetic divergence of the 5′-terminal *VP3* coding region. Moreover, no positively selected site was found in the VP1, VP2 and VP3 proteins. Additionally, no recombination of the *VP1*, *VP2*, and *VP3* genes was found in the studied strains. The exterior surfaces of the VP1, VP2, and VP3 proteins are rich in loops and are highly variable within the HRV-C population. The results suggested that the *VP1, VP2* and *VP3* genes, which encode major structural proteins of HRV-C, uniquely and rapidly evolved without positive selections.

Comprehensive molecular evolutionary and/or molecular epidemiologic studies of HRV-ABCs have been reported^13^,^14^. However, almost all studies were partially analyzed with regard to the *VP4*/*VP2* coding region of HRV^14^,^15^. The genes coding the VP proteins have many hypervariable regions; thus, it is difficult to design common primers for the amplification of the *VP1*, *VP2*, and *VP3* genes^12^,^16^. In addition, it may not be possible to isolate HRV-C using conventional methods at this time^2^. Thus, the antigenicity of HRV-C is still unknown. In the present study, we used an improved RT-PCR method with new primer sets and NGS, and we detected and analyzed the full length *VP1*, *VP2* and *VP3* genes with a high probability (>70%). To the best of our knowledge, this may be the first large study of the complete *VP1*, *VP2*, and *VP3* genes using many clinical strains.

Although HRV-C was recently discovered, it is thought that it may have a long history as a species. Indeed, Kiyota *et al*. showed that Japanese HRV-C strains could be dated back to the 1870s, according to the analysis of the *VP4/VP2* coding region^14^. The present strains dated back approximately 400 to 900 years (Fig. 1,2,3). Previous reports have suggested that HRV-C and HRV-A are frequently detected in patients with various ARIs^17^. Additionally, with regards to the analysis of the *VP4*/*VP2* coding region, both viruses exhibited large genetic divergence with many genotypes^15^. For example, Arakawa *et al*. showed that the *VP4/VP2* coding region in HRV-ABCs had over 0.3 divergence^17^. In the present study, greater than 0.3 divergence was found in the *VP1*, *VP2* and *VP3* genes. High genetic divergence in the 5′-terminal *VP3* coding region and the 3′-*VP1* coding region was found. The results from the partial analysis were compatible with our findings^12^,^18^. These results suggested that HRV-C might have a long history dating back at least 100 years; however, further studies are needed to confirm this.

The VP proteins of picornaviruses, which include HRV, play roles in their biology^7^. VP1, VP2, and VP3 protein are located at the surface of the viral capsid and are exposed to immune pressure, whereas VP4 is located inside the capsid^19^. For example, the VP1, VP2, and VP3 protein of many types of enteroviruses, such as EV71, is essential for the virus's ability to infect the host cells and acts as a protective protein in the viral shell^20^. Additionally, these proteins are recognized as major antigens in the host^20^. Indeed, the VP1 protein of many EVs, including HRV-A, is a major antigen^1^. However, the VP1, VP2, and VP3 proteins are major antigens for some types of HEVs^5^. It has been suggested that positive pressure in the host is associated with positive selection sites in major antigens^7^. Positive selection shows a survival advantage under the selective constraints that confront the viral population^7^. In the studied HRV-C strains, positive selection site was not found in the VP1, VP2 and VP3 proteins. Thus, HRV-C may be hardly affected under positive selection in our immune system.

Next, many negative selection sites (>100) were found in VP proteins in the present HRV-C strains. In general, negative selection plays an important role in maintaining the long-term stability of biological structures by removing deleterious mutations^7^. In the present study, many negative selection sites (>200) were found in both genes. Kiyota *et al*. showed that over 100 sites were found in the *VP4/VP2* coding region in HRV-C^14^. Thus, the negative selection sites in the VP1, VP2 and VP3 proteins may play the same roles as those in the *VP4/VP2* coding regions^21^,^22^.

Our *in silico* structural analysis disclosed that the exterior surfaces of the VP1, VP2, and VP3 proteins are rich in loops, highly variable within the HRV-C population. It is conceivable that the exterior loops contain neutralization epitopes of HRV-C. In contrast, the interior regions of the VP1, VP2, and VP3 proteins were less diverse, suggesting the presence of functional and/or structural constraints on the diversity of this region. Some sites within these regions may be important for interactions with the infection receptor or the formation of a functional capsid complex structure. However, further studies may be needed to determine whether the VP1 protein is the major antigen in the infective HRV-C strains.

In conclusion, HRV-C was detected in various ARI patients, and the virus exhibited large genetic divergence with a uniquely rapid evolution. Additionally, these viruses have been agents of ARI for a lot longer than previously thought.

## Methods

### Samples and patients

Nasopharyngeal swabs were collected from 2,922 patients with ARI between November 2007 and March 2013. ARI patients were diagnosed mainly with upper respiratory infection (URI) or lower respiratory infection (LRI; bronchitis, bronchiolitis, and pneumonia). The samples were obtained by the local health authorities of the Fukui prefecture, Kumamoto prefecture, Tochigi prefecture, and Yokohama Medical Center for the surveillance of viral diseases in Japan. Informed consent was obtained from the patients or their guardian for the donation of the samples.

### RNA extraction, RT-PCR and de novo sequencing by NGS

Viral RNA was extracted from 140 μL of supernatant using the QIAamp Viral RNA Mini Kit without carrier RNA (Qiagen, Valencia, CA). RT-PCR was performed using the PrimeScript® II High Fidelity One Step RT-PCR Kit (TaKaRa Bio, Otsu, Japan) and the primer pair HRV-C_546F: 5′- CTACTTTGGGTGTCCGTGTT -3′ and HRV-C_6410R: 5′- CCRTCATARTTDGTRTARTCAAA -3′. The PCR reactions are described in Suppl. Fig. S1. The NGS DNA library was prepared using a Nextera XT DNA sample prep kit (Illumina, San Diego, CA) with 96 indexing, followed by 200-mer paired-end *de novo* sequencing with MiSeq (Illumina). The obtained sequencing reads were assembled using the A5 assembler with the default parameters^23^.

### Phylogenetic analysis and estimation of the evolutionary rate using the Bayesian Markov chain Monte Carlo method

We aligned the nucleotide sequences of the *VP1, VP2* and *VP3* genes (positions; 2302-3126; 825 bp for HRV-QPM strain, positions 814-1602; 789 bp for HRV-QPM strain, positions 1603-2301; 699 bp for HRV-QPM strain) using CLUSTAL W [http://www.ddbj.nig.ac.jp/index-j.html]. To estimate the evolutionary rate and the time-scaled phylogeny, we used the Bayesian MCMC method in the BEAST package version 1.8.0^24^. The dataset was analyzed with a strict clock using the general time reversible with gamma-distributed rates across sites (GTR + I^−^) substitution model^25^,^26^ selected by the Kakusan4 program version 4.0 [http://www.fifthdimension.jp/products/kakusan/]^27^. The MCMC chain was run for 50 million steps to achieve convergence, with sampling every 1000 steps. Convergence was assessed by the effective sample size (ESS) after a 10% burn-in using the Tracer program version 1.6 [http://tree.bio.ed.ac.uk/software/tracer]. Only parameters with an ESS above 200 were accepted. Uncertainty in the estimates was indicated by the 95% highest posterior density (HPD) intervals. The maximum clade credibility tree was obtained using the Tree Annotator program version 1.8.0, and the first 10% of the trees were removed as burn-in. The phylogenetic tree was viewed in the FigTree program version 1.5 [http://tree.bio.ed.ac.uk/software/figtree/].

### Recombination analyses

Similarity plots showing the relationships between the aligned nucleotide sequences were generated using SimPlot, version 3.1 [http://sray.med.som.jhmi.edu/RaySoft/]^28^. The level of nucleotide similarity in each sequence, with a window size of 200 nt and a step size of 20 nt, was calculated using the Kimura 2-parameter method, and similarity plot analyses based on the deduced amino acid sequences of the VP2, VP3 and VP1 proteins were performed with a window size of 100 aa. In this analysis, 1 aa was calculated using the Kimura 2-parameter method. The sequences were applied to RDP 3 [http://darwin.uvigo.es/rdp/rdp.html] to predict the recombination events using RDP, GENECONV, BootScan, Maxchi, Chimaera, SiSscan and 3Seq^29^,^30^.

### Selective pressure analysis and the calculation of pairwise distances

To obtain estimates of the positively and negatively selected sites among the present strains during each season, we calculated the synonymous (dS) and nonsynonymous (dN) rates at every codon in the alignment using Datamonkey [http://www.datamonkey.org/]^31^. We used the following three different methods: single likelihood ancestor counting (SLAC), fixed effects likelihood (FEL), and internal fixed effects likelihood (IFEL). The SLAC and FEL methods were used to detect sites under selection at the external branches of the phylogenetic tree, while the IFEL method investigated sites along the internal branches. The SLAC method is best for large alignments but appears to underrate the substitution rate. Positively (dN > dS) and negatively (dN < dS) selected sites were determined by a *p*-value of <0.05 (SLAC, FEL, IFEL). Additionally, to assess the frequency distribution, we calculated the *p*-distance for the present strains, as previously described^10^.

### Molecular modeling of the HRV-C VP1, VP2, and VP3 proteins and analysis of amino acid diversity

Three-dimensional (3-D) models of the HRV-C VP1, VP2 and VP3 complex were constructed by homology modeling using ‘MOE-Align' and ‘MOE-Homology' in the Molecular Operating Environment (MOE) (Chemical Computing Group Inc., Quebec, Canada) as described for norovirus capsid protein modeling^32^,^33^. The X-ray crystal structures of HRV2 VP1 (PDB code: 3VDD), rhinovirus 14 capsid (PDB code:1R08) and human coxsackievirus VP3 (PDB code: 4GB3)^34^ were used as the modeling templates for HRV-C VP1, VP2, and VP3 proteins, respectively, because these templates exhibited high scores with low E-values. The HRV-C VP1, VP2, and VP3 models were superimposed on VP1, VP2, and VP3 in the complex containing VP1, VP2, VP3, and VP4 of HRV2 strain (PDB code: 3VDD). Amino acid diversity at individual sites in the HRV-C sequences obtained in this study was analyzed with Shannon entropy scores as previously described^32^.

### Ethical approval

The study was approved by the National Institute of Infectious Disease Ethics Committee (No. 495), and the study was conducted in compliance with the principles of the Declaration of Helsinki.

### Nucleotide sequence accession numbers

The sequences generated in this study have been assigned the GenBank accession numbers LC004772 to LC004910.

## Author Contributions

M.K., A.R., M.T., K.O., H.I. and H.K. designed research; S.N., T.S., H.T., M.Y. and M.N. performed research; H.S., N.K., K.S. and M.O. contributed analytic tools, S.N., T.S., H.T., H.S., N.K., K.K., K.S. and T.K. analyzed data; M.K., N.S., S.H. and H.K. wrote the paper.

## Supplementary Material

Supplementary Information Supplementary Information
