# Supplementary figures and images for: Molecular evolution of the VP1, VP2, and VP3 genes in human rhinovirus species C
Source: Sci Rep. 2015 Feb 2;5:8185. doi: 10.1038/srep08185 (PMC4313092; doi:10.1038/srep08185)

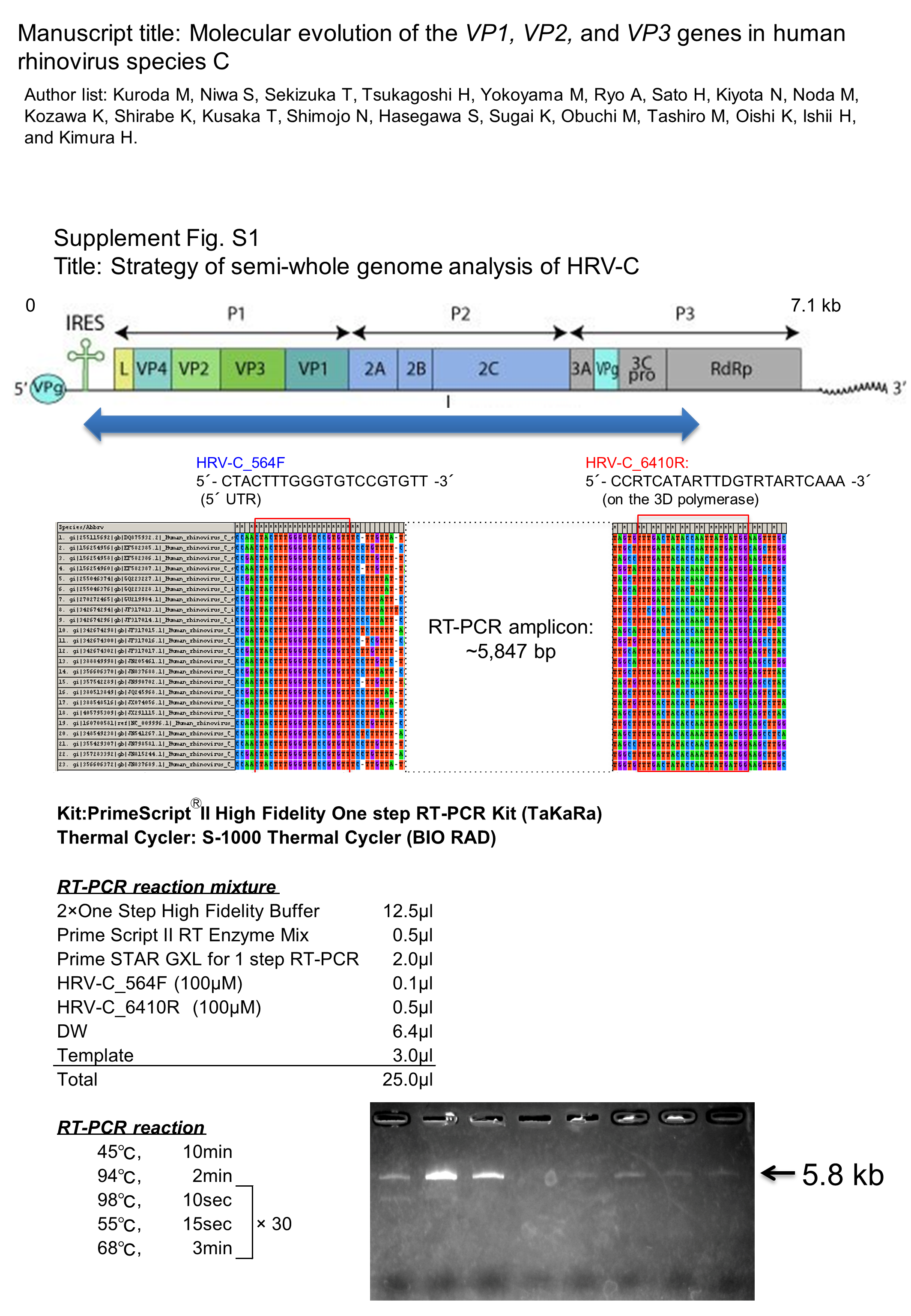

Supplement: Supplementary Information [file srep08185-s1.tiff]
